# Heparanase overexpression impedes perivascular clearance of amyloid-β from murine brain: relevance to Alzheimer’s disease

**DOI:** 10.1186/s40478-021-01182-x

**Published:** 2021-05-10

**Authors:** Xiao Zhang, Paul O’Callaghan, Honglian Li, Yingxia Tan, Ganlin Zhang, Uri Barash, Xiaomin Wang, Lars Lannfelt, Israel Vlodavsky, Ulf Lindahl, Jin-Ping Li

**Affiliations:** 1 Department of Neuroscience, Pharmacogly, University of Uppsala, The Biomedical Center Husargatan 3, Box 593, 751 23 Uppsala, Sweden; 2 Department of Medical Cell Biology, University of Uppsala, The Biomedical Center Husargatan 3, Box 571, 751 23 Uppsala, Sweden; 3 Stem Cell and Regenerative Medicine Lab, Institute of Health Service and Transfusion Medicine, Academy of Military Medical Sciences, Beijing, China; 4 Department of Medical Biochemistry and Microbiology, SciLifeLab Uppsala, University of Uppsala, The Biomedical Center Husargatan 3, Box 582, 751 23 Uppsala, Sweden; 5 Beijing Hospital of Traditional Chinese Medicine, Capital Medical University, No. 23, Back Road of Art Gallery, Beijing, 100010 China; 6 Cancer and Vascular Biology Research Center, The Rappaport Faculty of Medicine, 31096 Technion, Haifa Israel; 7 Department of Public Health and Caring Sciences, Rudbeck Laboratory, University of Uppsala, Molecular Geriatrics Dag Hammarskjölds väg 20, 751 85 Uppsala, Sweden

**Keywords:** Perivascular drainage, Heparan sulfate, Heparanase, Amyloid-β, Clearance, Aging, Alzheimer’s disease

## Abstract

Defective amyloid-β (Aβ) clearance from the brain is a major contributing factor to the pathophysiology of Alzheimer’s disease (AD). Aβ clearance is mediated by macrophages, enzymatic degradation, perivascular drainage along the vascular basement membrane (VBM) and transcytosis across the blood–brain barrier (BBB). AD pathology is typically associated with cerebral amyloid angiopathy due to perivascular accumulation of Aβ. Heparan sulfate (HS) is an important component of the VBM, thought to fulfill multiple roles in AD pathology. We previously showed that macrophage-mediated clearance of intracortically injected Aβ was impaired in the brains of transgenic mice overexpressing heparanase (Hpa-tg). This study revealed that perivascular drainage was impeded in the Hpa-tg brain, evidenced by perivascular accumulation of the injected Aβ in the thalamus of Hpa-tg mice. Furthermore, endogenous Aβ accumulated at the perivasculature of Hpa-tg thalamus, but not in control thalamus. This perivascular clearance defect was confirmed following intracortical injection of dextran that was largely retained in the perivasculature of Hpa-tg brains, compared to control brains. Hpa-tg brains presented with thicker VBMs and swollen perivascular astrocyte endfeet, as well as elevated expression of the BBB-associated water-pump protein aquaporin 4 (AQP4). Elevated levels of both heparanase and AQP4 were also detected in human AD brain. These findings indicate that elevated heparanase levels alter the organization and composition of the BBB, likely through increased fragmentation of BBB-associated HS, resulting in defective perivascular drainage. This defect contributes to perivascular accumulation of Aβ in the Hpa-tg brain, highlighting a potential role for heparanase in the pathogenesis of AD.

**Supplementary Information:**

The online version contains supplementary material available at 10.1186/s40478-021-01182-x.

## Introduction

A neuropathological hallmark of Alzheimer’s disease (AD) is amyloid plaques, the extracellular deposits of amyloid-β peptides (Aβ). These peptides, generally composed of 40 or 42 amino-acid residues, are generated by proteolytic cleavage of the Aβ precursor protein (AβPP) [18]. The “amyloid cascade hypothesis” posits that faulty clearance of Aβ from the brain contributes to the pathogenesis of sporadic AD, the predominant form of the disease (> 90% of cases) [12, 32]. Various Aβ clearance pathways have been proposed, such as Aβ phagocytosis by macrophages [15] and enzymatic degradation of Aβ by proteases [5]. Additionally, various blood-vessel associated clearance processes have been implicated, including receptor-mediated transport of Aβ across the endothelium into the blood [12], and perivascular drainage of Aβ along the vascular basement membrane (VBM) of capillaries and arteries [60]. Failure of these clearance pathways manifests as deposition of Aβ in the brain vasculature, known as cerebral amyloid angiopathy (CAA) [7, 12]. CAA is found in a number of neurodegenerative diseases including AD, but is also observed in human brains with no clear diagnosis of AD [2, 44, 54]. The molecular mechanisms behind these pathways are poorly understood.

Previous studies have implicated heparan sulfate proteoglycans (HSPGs) in various aspects of AD. HSPGs, ubiquitous on cell surfaces and in the extracellular matrix, consist of a core protein to which negatively charged heparan sulfate (HS) glycosaminoglycan chains are covalently attached [30]. HS is consistently found in Aβ plaques and CAA [6, 39], where it promotes aggregation of Aβ monomers [9, 22, 51]. Heparanase, an endo-β-glucuronidase that specifically degrades HS chains, is constitutively expressed in many tissues and can be transgenically overexpressed to elucidate the involvement of its polysaccharide substrate in various pathophysiological settings [57, 64]. We have previously shown that overexpression of heparanase in a transgenic mouse model (Hpa-tg) resulted in impaired macrophage-mediated clearance of intracortically injected Aβ [66]. Reduced Aβ deposition was noted when Hpa-tg mice were crossed with transgenic mice overexpressing the Swedish AβPP mutation [22]. Furthermore, selective elimination of neuronal HS reduces Aβ accumulation and promotes Aβ clearance through interstitial/perivascular drainage routes [31]. Together these findings indicate that heparanase, through its HS-degrading activity, can affect the balance between accumulation and clearance of Aβ in the brain.

Here we applied the Hpa-tg model to assess the involvement of HS and heparanase in Aβ accumulation along blood vessels. Clearance of injected aggregated human Aβ42 or endogenous murine Aβ was compromised in the Hpa-tg brain, as revealed by Aβ deposition in blood vessels and at distinct thalamic sites. Defective perivascular clearance was confirmed following intracortical injection of dextran, as a greater incidence and retention of vessel-associated dextran was detected in Hpa-tg brain compared to controls. Electron microscopy analysis revealed significant thickening of the VBM in Hpa-tg brain along with swelling of the perivascular astrocyte endfeet. Immunoblotting for the BBB-associated water pump protein aquaporin-4 (AQP4) detected elevated levels in Hpa-tg vs. control brains. These findings suggest that the observed defects in perivascular drainage are due to structural abnormalities at the blood–brain barrier (BBB), likely owed to excessive HS degradation by heparanase. Importantly, heparanase and AQP4 were also elevated in AD brain tissues, compared to non-demented controls, while heparanase activity was reduced in the CSF and plasma of AD. These findings highlight a role for heparanase in the perivascular clearance pathways in the brain, suggesting that heparanase may participate in Aβ clearance through degradation of HS and contribute to the pathogenesis of AD.

## Materials and Methods

### Mice

Transgenic mice overexpressing human heparanase on a C57BL/6 background (Hpa-tg) and non-transgenic Ctrl C57BL/6 mice were 3–20 months old [66]. Brain specimens from AβPP knock-out mice (strain B6.129S7-APPtm1Dbo/J, The Jackson Laboratory) were kindly provided by Dr. Lars NG Nilsson (Department of Pharmacology, University of Oslo and Oslo University Hospital). All experiments were approved by the regional animal research ethics committee (C165/15, Uppsala, Sweden).

### Human brain tissues, cerebrospinal fluid and plasma

Postmortem specimens of medial temporal gyrus from 8 AD individuals (88 ± 3.6 year-old) diagnosed with Braak stage V-VI AD pathology and 8 age-matched (84 ± 3.7 year-old) non-demented Braak stage I-II controls were obtained from the Netherlands Brain Bank (NBB), Netherlands Institute for Neuroscience, Amsterdam. The average postmortem delay time of the brain tissues was 6.2 ± 1.7 h (Additional file 1: Table 2). Thalamic tissue sections from two sporadic AD patients were obtained from Dr. Martin Ingelsson. The experiments were approved by the regional ethical committee (2005–103, 2005–06-29, Uppsala Sweden). Additionally, cerebrospinal fluid (CSF) and plasma from 27 AD cases (84 ± 4.9 years-old) and 14 non-demented control cases (89 ± 10 years-old) were obtained from the NBB. All donors gave written informed consent for brain autopsy and the use of their specimens and medical records for research purposes. All AD samples were derived from individuals diagnosed with Braak stage AD pathology between IV and VI (Additional file 1: Table 3).

### Intracerebral injections of Aβ42 and dextran

Aβ42 aggregates more rapidly than Aβ40 [49], and was thus chosen for this study. Injections were performed on deeply anesthetized (2.5% Avertin, 500 µl/mouse i.p.) mice under stereotaxic guidance with coordinates from the bregma: + 2.0 mm anteroposterior, − 2.0 mm lateral, and − 2.3 mm dorsoventral for Aβ42; and in another group of mice from bregma: + 2.0 mm anteroposterior, − 1.8 mm lateral, and − 1.6 mm dorsoventral for dextran. For Aβ42 injection, 5 µg of aggregated synthetic human Aβ1-42 (PolyPeptide Laboratories GmbH, Germany) (5 µg/1 µl) was injected into one hemisphere at a rate of 0.2 µl/min, followed by a 2-min pause for absorption of the injected solution. The mice were sacrificed 1, 2, and 4 weeks after the intracerebral injection. Fluorescent dextran (Tetramethylrhodamine, 10,000 MW, fluoro-Ruby, Life Technologies) was dissolved in saline at a concentration of 1 mg/ml. Each animal received 0.7 µl of the dextran preparation, administered as described above for the Aβ42 injections. At the time of sacrifice, deeply anesthetized mice were transcardially perfused with 50 ml saline and the brains were recovered, fixed in 4% formaldehyde overnight and processed according to standard protocols for preparation of paraffin sections and frozen sections.

### Western blotting

Protein samples were separated by 10–20% SDS-PAGE and then transferred to a nitrocellulose membrane. After blocking with 5% nonfat dry milk, the membranes were probed with primary antibodies (Additional file 1: Table 1) followed by the corresponding secondary antibodies. Signals were visualized using SuperSignal West Pico or Dura substrates (Thermo). Quantitative band analysis was performed using ImageJ software.

### β-Secretase (BACE1) activity assay

Brains of Hpa-tg and Ctr mice were dissected and the cortex, hippocampus and thalamus were separated and homogenized in CelLytic™ MT (Sigma) containing protease inhibitors (Roche). The homogenates were centrifuged at 20,100xg for 1 h at 4 °C. The supernatants were collected and subjected to BACE1 activity assay using the SensiZyme BACE1 assay kit (Sigma).

### Immunostaining and histochemistry

Immunostaining and Congo red histochemistry were performed on 5 μm paraffin sections. Antigens were retrieved in 25 mM citrate buffer (pH 7.2) or Rodent Decloaker (Biocare Medical, USA) using a microwave oven. Sections were blocked with M.O.M.™ IgG block (Vector Labs, USA) or Rodent Block M (Biocare Medical). Primary antibody (Additional file 1: Table 1) incubation was carried out overnight at ~ 4 °C followed by incubation with secondary antibody for 30–60 min at room temperature. ABC™ complex and NOVA™ red reagents (Vector Labs) were used to visualize the immunosignals. In other settings, MM AP-Polymer kit (mouse antibody on mouse tissues) or MACH3™ Rabbit-Probe Alk Phos Polymer kits were employed along with Vulcan Fast Red Chromogen kit 2 (Biocare Medical). For double immunostaining using fluorescent secondary antibodies, primary antibodies were incubated overnight, simultaneously or stepwise at 4 °C, followed by incubation with the appropriate secondary antibodies (Alexa Fluor, fluorescent secondary antibodies against mouse and rabbit, Invitrogen). DAPI (4′,6-diamidino-2-phenylindole) was used for counterstaining of nuclei. Sulfated alcian blue staining was performed according to Lendrum et al. [28].

### Microscopy and image analysis

Microscopy was performed using a Nikon DXM1200F™ instrument (Nikon, Melville, USA). Z-stacks for selected deposits were obtained by confocal laser scanning microscopy using a Carl Zeiss LSM 510 Meta instrument (Carls Zeiss, Germany). Three-dimensional images were created and analyzed with Imaris imaging software (BitplaneAG, Switzerland). The mean dextran fluorescence associated with αSMA (α-smooth muscle actin)-positive vessels was determined as follows: αSMA-positive vessels were outlined as regions of interest (ROI) using ImageJ software. These ROIs were overlaid on the image’s dextran fluorescence channel and the average fluorescence of vessel-associated dextran (F^V.Dx^) was measured and recorded using ImageJ. At least four additional ROIs (in areas that were negative for αSMA) were identified in each image and the mean value for the average fluorescence of dextran in the background (F^Bg.Dx^) was calculated. Only vessels with F^V.Dx^ greater than F^Bg.Dx^ were considered to be dextran positive. This method was used to determine the number of dextran positive vessels in brain sections from n = 8 Hpa-tg mice and n = 6 Ctrl mice. The average dextran intensity analysis was only performed on vessels determined to be dextran-positive, a total of n = 33 vessels from Hpa-tg brain sections and n = 25 vessels from Ctrl brain sections were compared.

### Transmission electron microscopy

4-month-old Hpa-tg and Ctr mice were euthanized by CO_2_, and the entire brains were dissected immediately after death. The brains were fixed in 2% glutaraldehyde in 0.1 mol/L sodium cacodylate buffer supplemented with 0.1 mol/L sucrose. The frontoparietal cortex was microdissected. Semithin sections (0.5 µm) were cut and stained in 1% v/v toludine blue and the stained sections were used as a guide to cut ultrathin 80 nm sections of areas containing capillaries. The ultrathin sections were collected onto copper grids which were covered with a film of polyvinyl formal plastic and contrasted with uranyl acetate and lead citrate. Electron micrographs of capillaries were taken with a Hitachi electron microscope (Hitachi Ltd, Tokyo, Japan). The thickness of VBM and the size of perivascular astrocyte end-feet from capillary cross-sections were assessed with the NIH ImageJ software. The thickness of VMB was calculated by dividing the VBM area by capillary circumference (Additional file 2: Fig. 3). The size of perivascular astrocyte end-foot was calculated by subtracting the blood vessel area (including the BM; Additional file 3: Fig. 4a: the area within the yellow curved line) from the area enclosed by the outer edge of the astrocyte end-feet, designated total capillary area (Additional file 3: Fig. 4a: the area within the blue curved line), and was expressed as percentage of the total capillary area.

### Heparanase enzymatic activity assay

Heparanase activity in CSF and plasma samples of AD and non-demented control cases was determined using homogeneous time-resolved fluorescence (HTRF) technology based on the capacity of a known concentration of heparanase to degrade biotin and europium cryptate labeled HS (Biotin-HS-Eu) (Cisbio, Marcoule, France) [13]. Serially diluted purified recombinant human heparanase (PMID: 23,162,016) was used to set up the standard curve. CSF and plasma samples were incubated with streptavidin-conjugated magnetic beads (Sigma-Aldrich) at 4 °C for 48 h to remove biotin-conjugated molecules. Ten microlitres of each sample solution or blank buffer (50 mM Tris–HCl pH 7.4, 0.15 M NaCl, 0.1% protease-free BSA, 0.1% CHAPS) and 4.2 ng of the Biotin-HS-Eu substrate in 5 µl of 0.2 M Acetate buffer pH 5.5 were added to a 384-well low volume microplate and incubated at 37 °C for 30 min. Twelve nanograms of streptavidin-d2 acceptor (Cisbio, Marcoule, France) in 5 µl of 62.5 mM Hepes pH 7.4, 0.8 M KF, 0.1% protease-free BSA, and 2 mg/ml heparin were added to stop the reaction. After 15 min incubation at RT, the heparanase enzymatic activity was detected by determining the HTRF signals with a FLUOstar Omega plate reader (BMG Labtech, Germany), and the heparanase activity was then calculated from the standard curve, done by BMG LABTECH MARS data analysis software installed in the plate reader. Each reaction was run in duplicate.

### Statistical method

Two-tailed unpaired Student's t-test was used to determine the statistical significance of differences between population means. Statistical significance was set at P < 0.05.

### Results

### Accumulation of intracortically injected human Aβ42 in the thalamus of Hpa-tg mice

**Fig. 1:**
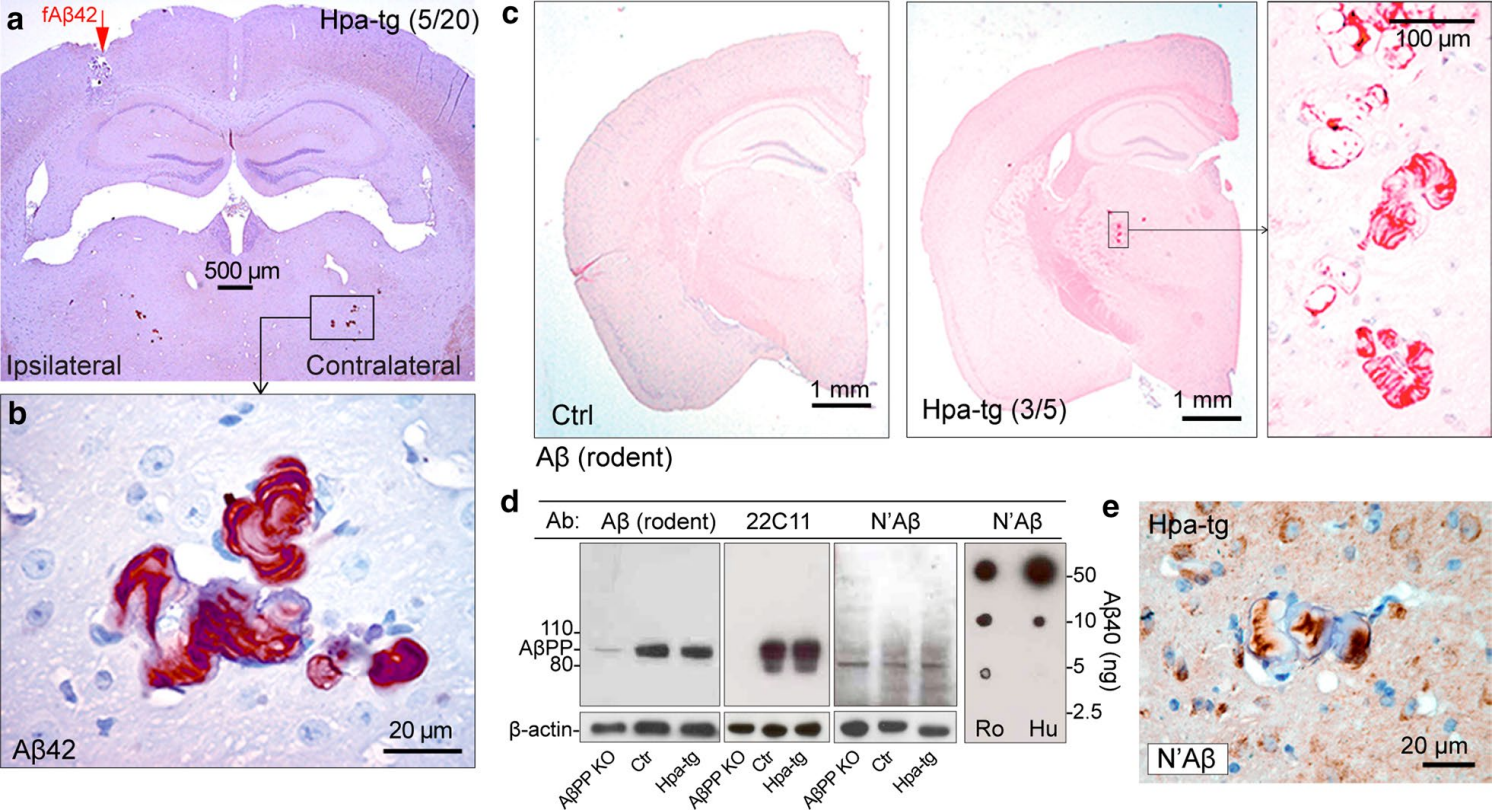
Thalamic accumulation of Aβ in Hpa-tg mice. (**a, b**) Translocation of intracortically injected Aβ42 to the thalamus of Hpa-tg mouse. (**a**) Anti-Aβ42 immunostaining of a Hpa-tg mouse brain four weeks after intracortical injection (*red* arrow in a) of aggregated synthetic human Aβ42. (**b**) Enlarged view of a thalamic Aβ42 deposit with layered morphology. (**c-e**) Thalamic accumulation of endogenous murine Aβ in Hpa-tg (> 14-month-old) mice. (**c**) Anti-rodent Aβ immunostaining of 14-month-old Ctr (left panel) and Hpa-tg (middle panel) brains. Enlarged view of a layered thalamic Aβ deposit in Hpa-tg brain, is shown in the right panel. (**d**) Western blotting analyses of brain homogenates from AβPP knockout (AβPP KO), Ctr and Hpa-tg mice applying antibodies directed against rodent Aβ, AβPP (22C11), and the N-terminal domain of human Aβ (N’Aβ). Dot-blot assay of synthetic human and rodent Aβ40 detected with the anti-N’Aβ antibody, is presented in the right panel. (**e**) Immunostaining with anti-N’Aβ antibody detects a layered deposit of endogenous Aβ in the thalamus of Hpa-tg mouse

We previously reported the retention of intracortically injected synthetic human Aβ42 in the brains of Hpa-tg compared to Ctrl mice [66]. Here, we investigated the mechanisms behind this phenomenon. Immunostaining of the brain sections with anti-Aβ42 antibodies specific for the C-terminal epitope (anti-Aβ42) detected strong signals in the thalamic ventral posterior nuclei (VPN), in 5 of the aged animals (≥ 14 month-old) out of 20 injected Hpa-tg mice (Fig. 1a and b, and Additional file 4: Fig. 1a and b), but not even in a single one of the 18 injected Ctrl mice (Additional file 4: Fig. 1i and j). The thalamic Aβ42 deposits showed a distinct layered morphology (Fig. 1b) and were detected in both ipsilateral and contralateral VPN (Fig. 1a). Close examination of the sections of Hpa-tg mouse brain also revealed Aβ42 immunosignals in the cortical interstitial spaces close to the injection site (Additional file 4: Fig. 1b and c), and associated with blood vessels in the hippocampus (Additional file 4: Fig. 1b and d), cortex (Additional file 4: Fig. 1e) and thalamus (Additional file 4: Fig. 1f). The layered morphology of the thalamic Aβ deposits is reminiscent of the “vessel within vessel” appearance of Aβ deposition in the blood vessel wall previously described [45]. Double immunostaining, with anti-vWF antibody for blood vessels and anti-Aβ 6E10 antibody, confirmed these deposits to be associated with vessels (Additional file 4: Fig. 1g and h). These findings suggest that the injected Aβ42 was translocated along, and accumulated within perivascular drainage pathways that connect the injection site to the thalamus.

### Thalamic deposition of endogenous Aβ in Hpa-tg mice

**Fig. 2:**
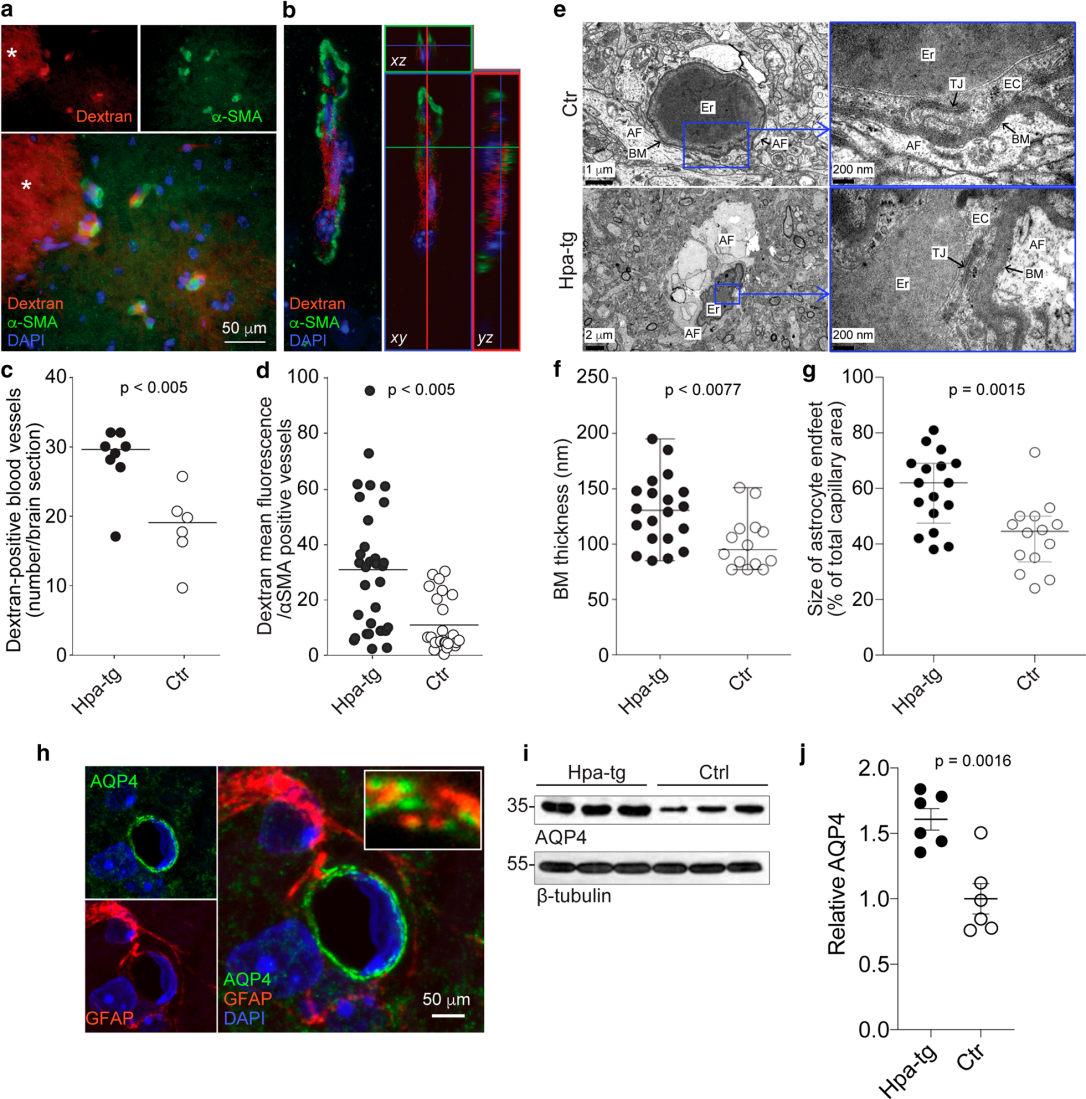
Impaired perivascular drainage and altered BM structure in Hpa-tg mice. (**a-d**) Vascular retention of intracortically injected fluorescent dextran in Hpa-tg and Ctrl brains. (**a**) Association of dextran (*red*) with α-SMA positive blood vessels (*green*) in the proximity of the injection site indicated by an asterisk (*). Cell nuclei were counterstained with DAPI (*blue*). (**b**) Confocal laser-scanning microscopy image of dextran- and α-SMA positive blood vessel. (**c**) Number of dextran-positive blood vessels (defined by α-SMA immunostaining) per brain section. Each point represents the data from an individual mouse brain (n = 6 Ctr mice, n = 8 Hpa-tg mice). (**d**) Mean dextran fluorescence associated with αSMA-positive blood vessels. Each point represents the dextran fluorescence measured from an individual blood vessel (n = 25 vessels, Ctr mice; n = 33 vessels, Hpa-tg mice). (**e–g**) Thickening of the VBM and swelling of the perivascular astrocyte endfeet in the cortex of Hpa-tg brain. (**e**) Electron micrographs of capillaries from Ctr and Hpa-tg brain sections. Enlarged views of Ctr and Hpa-tg images illustrate the positions of the basement membrane (BM), endothelial cell (EC) with tight junction (TJ), astrocyte endfoot (AF), and erythrocyte (Er) within the vessel. (**f**) VBM thickness analysis. (**g**) Size of perivascular astrocyte endfeet presented as percentage of the total capillary area. (**h**) Aquaporin 4 (AQP4) and glial fibrillary acidic protein (GFAP) immunostaining associated with a mouse brain vessel. (**i**) AQP4 Western blots of Hpa-tg and Ctrl brain homogenates. (**j**) Quantification of the relative AQP4 Western blot bands from Hpa-tg (n = 6) and Ctrl (n = 6) brain homogenates, corrected for β-tubulin loading controls

During efforts to determine the potential contribution of endogenous rodent Aβ to the thalamic deposits observed in the injected Hpa-tg brains, non-injected Hpa-tg brain sections were immunostained with an anti-rodent Aβ antibody. Unexpectedly, immunopositive deposits were found in the VPN of 3/5 aged (≥ 14 months) Hpa-tg mice, but not in age-matched control mice (n = 5) (Fig. 1c). The deposits were similar in appearance to those observed in Hpa-tg mice injected with human Aβ42 (as seen in Fig. 1b). Additionally, the thalamic deposits in the Aβ-injected and the non-injected Hpa-tg mice stained positive for sulfated Alcian blue (SAB), indicating the co-accumulation of HS at these sites, but negative for Congo red (Additional file 5: Fig. 2a and b), suggesting that these deposits lack β-sheet secondary structure. However, while the thalamic deposits in the Aβ-injected mice were readily detected with an anti-Aβ42 antibody (Fig. 1a and b), the deposits in the non-injected mice were not detected by anti-Aβ40 or anti-Aβ42 antibodies specific for C-terminal epitopes (Additional file 5: Fig. 2b). Murine and human Aβ sequences are identical between residues 14–42 [61], suggesting that the antibodies would detect such epitopes if present. Western blotting of brain homogenates from control and Hpa-tg mice indicated that the anti-rodent Aβ antibody detected a band equal in molecular weight to that detected by the AβPP-specific antibody 22C11, which, as expected, was absent from AβPP knock-out mice (Fig. 2d). This suggests that the thalamic deposits in non-injected Hpa-tg mice may in part be composed of AβPP, or fragments thereof. To further probe the molecular identity of the thalamic deposits in Hpa-tg mice we used an Aβ antibody specifically recognizing an N-terminal epitope common to Aβ40, 42 and 43 (anti-human Aβ [N’Aβ]). Western blotting revealed that this antibody did not detect AβPP, but cross-reacted with rodent Aβ40 as shown by dot-blot analysis (Fig. 1d). Similar to the anti-rodent Aβ antibody, the N’Aβ antibody revealed Aβ deposits with layered morphology in the VPN of Hpa-tg mice (Fig. 1e). Notably, AβPP levels and activity of the AβPP-cleaving enzyme BACE1 were similar in Ctrl and Hpa-tg brain (Additional file 5: Fig. 2c–e), suggesting that altered AβPP metabolism was not a contributing factor to the accumulation of endogenous Aβ in Hpa-tg thalamus. We conclude that these thalamic deposits in Hpa-tg brain consist of endogenous Aβ peptide, which is likely C-terminally truncated.

### Delayed perivascular clearance of injected dextran in Hpa-tg brain

The thalamic Aβ deposits in both Aβ injected and non-injected Hpa-tg brains resembled endogenous Aβ deposits previously reported in the VPN of rats following transient occlusion of the middle cerebral artery [52], attributed in part to defective perivascular clearance along interstitial drainage routes. To elucidate whether the Aβ accumulation is a property of the peptide, we assessed the association of fluorescent dextran with α-SMA immunopositive blood vessels, 15 min after intracortical injection of dextran (Fig. 2a and b). The incidence of dextran-associated vessels was significantly more frequent in Hpa-tg than in Ctrl mice (Fig. 2c), as was the mean dextran fluorescence associated with α-SMA-positive blood vessels (Fig. 2d), indicating greater dextran accumulation in the vasculature of Hpa-tg vs. Ctrl brain. A similar defective clearance of dextran was observed in a Tg2576 AD mouse model and attributed, in part, to compromised perivascular drainage due to the presence of cerebral amyloid angiopathy [20].

### Altered ultrastructure of capillaries and increased expression of aquaporin 4 in Hpa-tg brain

Because HSPGs, including perlecan and agrin, are major components of the VBM and closely associated with capillary structure and functions, we sought to investigate the effects of heparanase overexpression on the ultrastructure of the VBM. Quantitative analysis of transmission electron microscopy images of cerebral capillaries revealed the VBM to be significantly thicker (Fig. 2e and f), and astrocyte endfeet to be significantly larger (Fig. 2e and g) in Hpa-tg mice compared to controls. The latter was indicative of astrocyte endfeet swelling.

Aquaporin 4 (AQP4) is a water-selective membrane transport protein, which localizes to astrocyte endfeet at the BBB [62] (Fig. 2h) and glial AQP4 expression is upregulated in brain pathologies including trauma and inflammation [17, 33, 62]. Furthermore, AQP4 expression can be regulated by the extracellular HSPG agrin [35]. Analysis of AQP4 immunoblots revealed significantly elevated levels in Hpa-tg compared to control brains (Fig. 2i and j).

### Elevated levels of heparanase and aquaporin 4 in AD brain

**Fig. 3:**
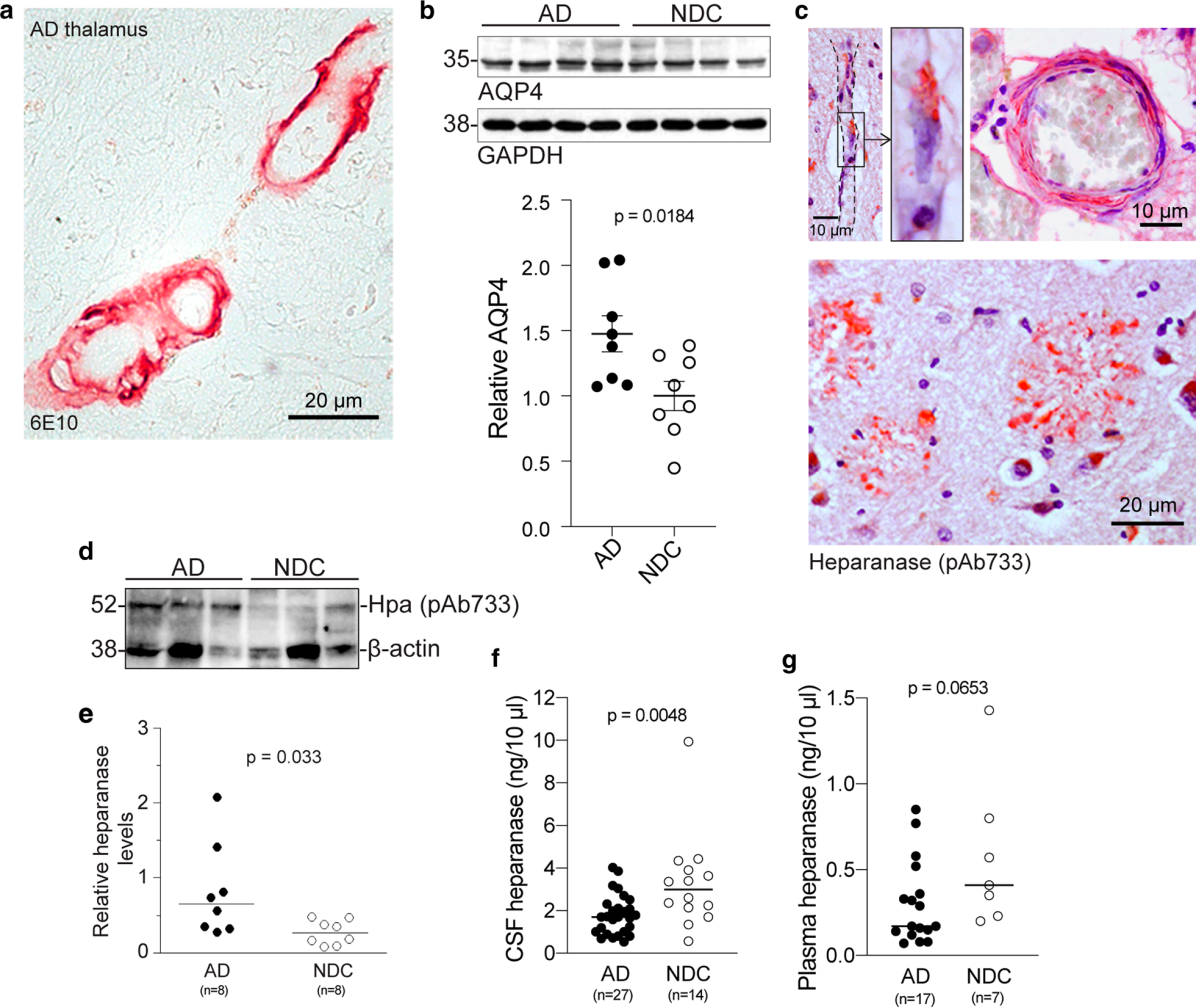
Elevated levels of AQP4 and heparanase in AD brain. (**a**) Aβ42 immunostaining in the thalamus of AD brain. (**b**) AQP4 Western blotting and quantitative analysis of relative band intensities, corrected for GAPDH loading controls, of AD and non-demented control (NDC) medial temporal gyrus homogenates. (**c**) Heparanase (pAb733) immunostaining in the hippocampus of AD brain. The upper left panels illustrate heparanase distribution in microvessels, with an enlarged view in the upper centre panel. The upper right panel illustrates heparanase distribution in an arteriole. The lower panel reveals heparanase immunostaining that reveals Aβ-deposit like morphology within AD hippocampus (see also Suppl. Figure 5). (**d**) Heparanase (pAb733) Western blotting and (**e**) quantitative analysis of relative band intensities, corrected for β-actin loading controls, in homogenates from AD and NDC medial temporal gyrus. (**f, g**) Heparanase activity in cerebrospinal fluid (CSF) and plasma of AD and NDC as determined by the homogenous time-resolved FRET (HTRF) assay

While Aβ accumulation as CAA is often observed in AD, the thalamus is not the most common site for these lesions, which are typically localized to the cortex and leptomeninges [10]. Nonetheless, with Aβ42 immunostaining we could detect vascular deposits in AD thalamus (Fig. 3a). Morphological aberrations of cerebral VBM in AD have been previously described [14], and elevated AQP4 levels in AD have been reported [33]. Here, applying human brain specimens of medial temporal gyrus, we provide further evidence showing elevated AQP4 levels in AD compared to age-matched non-demented control (NDC) (Fig. 3b), similar to the observation in Hpa-tg brain (Fig. 2i and j). The AD hippocampus showed various instances of heparanase-associated vessels, including microvessels (Fig. 3c, upper left and central panel) and leptomeningeal arterioles (Fig. 3c, upper right panel). In addition, heparanase immunostaining presented with Aβ deposit morphology in AD hippocampus (Fig. 3c, lower panel, and Additional file 6: Fig. 5). Western blotting revealed significantly higher level of heparanase in homogenates of the medial temporal gyrus of AD compared with age-matched NDC (Fig. 3d and e).

### Decreased heparanase activity in AD CSF and plasma

Finally, we measured heparanase activity in CSF and plasma using the HTRF technique [13]. Heparanase activity was detected in all analyzed CSF samples (n = 27 AD and n = 14 NDC) but was not detected in the plasma of 10 out of 27 AD and 7 out of 14 NDC samples. Heparanase activity was significantly lower in AD CSF compared with NDC CSF (Fig. 3f), and similarly, lower levels were detected in AD plasma compared with NDC plasma (Fig. 3g).

## Discussion

Heparanase has been implicated in various pathological conditions, in particular cancer and inflammatory diseases [29, 55, 65]. The pathophysiological effects of the enzyme are generally interpreted in terms of degradation of HS chains, although the enzyme may exert non-catalytic activities, apparently through receptor activation and signal transduction [43, 56]. Our previous finding of inhibited clearance of injected aggregated Aβ42 in heparanase overexpressing murine brain was ascribed to degradation of endothelial cell-surface HS, resulting in impaired transmigration of blood-borne monocytes into the parenchyma, and lack of macrophages capable of Aβ uptake and degradation [66]. Transgenic overexpression of heparanase can thus be used to reveal functional effects of the heparanase-HS axis in pathophysiological settings We have now applied this system to provide insights into the elimination pathway of injected Aβ that had not been degraded in situ. Immunohistochemical visualization of injected human Aβ42 in Hpa-tg mice revealed an elimination pathway based on perivascular drainage of interstitial fluid (ISF) (Additional file 4: Fig. 1). No residual Aβ was detected along blood vessels of Ctrl mice, pointing to a compromised drainage system in the Hpa-tg animals. Deposits in the cortical vasculature and thalamus showed a compact nodular morphology (Fig. 1 and Additional file 4: Fig. 1), which has been referred to as a typical feature of perivascular drainage of Aβ [59, 60]. Similar deposits, but of endogenous Aβ, were seen in aged Hpa-tg, but not in age-matched Ctrl mice (Fig. 1), and compromised solute clearance via ISF pathways was confirmed in Hpa-tg mice by injection of Dextran (Fig. 2). Perivascular fluid drainage along the VBM of cerebral capillaries and arteries is a route by which proteins and other solutes are normally eliminated from the brain [8], and serves as a major pathway for removing Aβ from the brain [21]. This suggests that the thalamic accumulation of Aβ from the injection site or endogenous sources, is likely due to impaired perivascular drainage, resulting in vascular deposition of Aβ.

It has been proposed that vessel pulsations supply the motive force for perivascular drainage and that the VBM is essential in mediating such force [7, 60]. BMs are thin sheets composed of specialized proteins (collagen IV and laminin-entactin/nidogen complex), HSPGs (perlecan and agrin) and non-collagenous glycoproteins [23, 41]. Based on our identification of degraded HS in Hpa-tg mice, primary endothelial cells [66] and astrocytes [37], we considered that the impaired perivascular drainage may be due to a loss of HSPG function in brain capillaries. Accordingly, ultrastructural TEM analysis revealed significant thickening of the VBM and swelling of perivascular astrocyte end-feet in Hpa-tg compared to Ctrl brain (Fig. 2). Age-related VBM thickening in mice similarly correlates with impaired drainage of injected soluble Aβ40 from the hippocampus [19]. Importantly, VBM thickening and vacuolation are also observed in AD brain, associated with microangiopathy [42, 63], perturbed solute elimination from the parenchyma, and CAA development [20]. Notably, heparanase activity increases as an acute response in models of ischemic stroke and sepsis, and promotes degradation of the endothelial glycocalyx [27, 47] which in turn increases immune cell infiltration and associated inflammation. We attributed the impaired recruitment of macrophages to the Aβ injection site in the Hpa-tg brain to the loss of endothelial HS-binding sites [66], resulting from the constitutive overexpression of heparanase. Here we identify further defects in the Hpa-tg VBM, which may also play a role in impeding immune cell infiltration across the blood brain barrier.

In brain regions affected by AD, the endothelial surface of the VBM shows disturbed HSPG staining pattern [42]. The two HSPG species, perlecan and agrin, potentially implicated in the microstructural changes of Hpa-tg capillaries are likely targeted by heparanase. While perlecan is crucial for maintaining BM integrity [11, 26] and carries the HS chains that directly interact with collagen IV [36], agrin is involved in the polarized expression pattern of AQP4 in astrocyte endfeet [35]. AQP4 is the most abundant water-selective membrane transport channel in the brain [53] and is primarily localized to the plasma membrane of astrocytes apposed to pial or perivascular basal laminae [3, 4, 34, 40]. The observed swelling of perivascular astrocyte endfeet in Hpa-tg brain is indicative of altered water homeostasis, in accord with the elevated levels of AQP4 in Hpa-tg compared to Ctrl mouse brain (Fig. 2j). Importantly, AQP4 is upregulated in a variety of brain pathologies, including AD [17, 33, 62], as further supported in the current study (Fig. 3b). Upregulated vascular expression of heparanase resulting in HS degradation may also impair mechanisms of receptor-mediated clearance of Aβ, in which HS has been attributed co-receptor functions [24, 38]. While the exact mechanism behind the impeded perivascular solute drainage in Hpa-tg brain has yet to be fully elucidated, it seems reasonable to conclude that heparanase-mediated fragmentation of HS chains that are central to the structural organization, molecular composition and function of the capillary VBM, contribute substantially to the observed defects.

The reason behind the localization of Aβ deposits to the ipsilateral and contralateral Hpa-tg thalamus is not readily apparent. The thalamic deposits may mark a site of Aβ exit from the ISF into the bloodstream, as evidenced by their appearance following transient occlusion of the middle cerebral artery in rats [52]. While we could detect examples of vascular Aβ deposition in human AD thalamus (Fig. 3a) the pathophysiological significance of thalamic Aβ deposits remains unclear. Thal et al. found thalamic Aβ deposits mainly associated with later stages of the disease [50]. However, recent evidence from post-mortem studies, non-invasive imaging, and genetically modified animal models suggests that the loss of episodic memory in early AD involves the limbic thalamus, such that thalamic abnormalities occur in the early stages of AD (for review see [1]). Further work is required to establish whether such deposits contain material translocated from other brain regions.

Importantly, the phenomenon observed in the mouse model is also detected in AD brain, where heparanase levels were higher compared to NDCs, and immunostaining located the enzyme in blood vessel walls and in Aβ deposits (Fig. 3d). Notably, we found significantly lower heparanase activity in CSF and plasma of AD than NDC individuals. Heparanase is normally expressed at extremely low levels, but upregulated under pathological conditions, e.g., cancer and inflammation, as a reactant protein. Our earlier study revealed that overexpressing heparanase attenuated Aβ plaque formation in mice overexpressing the Swedish AβPP mutation [22], signifying that heparanase degradation of HS prevented Aβ deposition. Indeed, elevated heparanase transcript expression was detected in AD brain [16], suggesting that heparanase accumulation is at least in part due to increased enzyme production. Thus, the excess deposition of heparanase along with Aβ in both aged Hpa-tg and human AD brain may imply an active involvement of heparanase. Unexpectedly, decreased activity of heparanase was detected in the CSF and plasma of AD. We do not know whether the low heparanase enzymatic activity in AD samples reflects inactivation of the enzyme or a reduced quantity, as the amount of protein was under the detection limit of the currently available methods. Nevertheless, this result demonstrate that the HTRF assay is sensitive enough to measure heparanase activity in body fluids as a potential AD marker.

Our results point to a link between increased expression of heparanase and the development of CAA in AD. They extend the pathophysiological roles of HSPGs in Aβ pathology from aggregation/deposition and cytotoxicity [25, 39, 46, 48, 58], to clearance through Aβ phagocytosis [66], and elimination via the perivascular drainage pathway. It remains to be determined whether altered heparanase expression and/or activity occur as a response to the pathophysiological changes in the AD brain or is causally involved in the progression of the disease. Nonetheless, through its HS-degrading capacity, heparanase can modulate various processes involved in the pathogenesis of AD and hence warrants further studies as a potential novel drug target.

## Supplementary Information

**Additional file 1.** Supplementary Table 1: Primary antibodies used in this study. Supplementary Table 2: The middle temporalis gyrus of Alzheimer’s disease and Non-demented control obtained from the Netherlands Brain Bank (NBB). Supplementary Table 3: The CSF and plasma samples of Alzheimer´s disease patients and non-demented controls from the Netherlands Brain Bank (NBB).

**Additional file 2.** Thickness of vascular basement membrane (VBM). The VBM area was calculated using ImageJ software as the area enclosed by the blue curved line minus the area within the yellow curved line. Subsequently, the relative thickness of the VBM was calculated by dividing the VBM area by the capillary perimeter, i.e. the length of the blue curved line. The results were then expressed as BM thickness (nm) in Fig 2f. AF: astrocyte endfeet. E: erythrocyte.

**Additional file 3.** Swelling of astrocyte endfeet in Hpa-tg brain revealed by transmission electron microscopy. a) Image analysis was conducted using ImageJ software. The sums of the area of the astrocyte endfoot and the blood vessel-specific area were designated as the total capillary area, defined by the curved blue line. Thus the astrocyte endfoot area equaled the total capillary area minus the blood vessel-specific area (enclosed by the curved yellow line), which was then expressed as percentage of the total capillary area (Fig. 2g). b-g) Representative micrographs obtained from two mice of each strain (Hpatg and Ctr); b-d) Ctr. e-g) Hpa-tg. AF: astrocyte endfoot, BM: basement membrane, E: erythrocyte, EC: endothelial cell, TJ: tight junction.

**Additional file 4.** (a) Schematic illustration of mouse brain regions taken from the Allen Brain Atlas and identifying the location of the ventral posteromedial nucleus (VPL) and ventral posterolateral nucleus (VPM) of the thalamus, collectively referred to as ventral posterior nuclei (VPN). (b) Aβ42 immunostaining of a Hpa-tg brain section derived from a mouse that received an intracortical injection of fibrillar Aβ42 (fAβ42). (c) Enlarged view of a region close to the injection site in which Aβ42 was detected in interstitial spaces (arrows) and associated with blood vessels (arrowhead). (d) Enlarged view of a hippocampal region illustrating perivascular Aβ42 immunosignals. (e) Aβ42 immunosignals associated with cortical vasculature (arrows). (f) Aβ42 deposition in thickened VPN blood vessel wall. (g) Confocal microscopy of a thalamic deposit immunostained with anti-vWF for blood vessels (green) and the anti-Aβ antibody 6E10 (red). (h) Reconstructed three-dimensional rendering of one of the vessels in (g). (i) Aβ42 immunostaining of a control brain section from a mouse that had received an intracortical injection of fibrillar Aβ42 (fAβ42). (j) Enlarged view of the thalamic region in a Ctrl brain section.

**Additional file 5.** (a) Sulfated Alcian blue (SAB) and Congo red (CR) histochemical staining of the thalamic Aβ deposits in an Aβ-injected Hpa-tg mouse. (b) SAB and CR histochemical staining of the thalamic Aβ deposits in Hpa-tg brain sections from a mouse that had not been injected with Aβ (upper panels). Immunostaining of the thalamic structures in non-injected Hpa-tg mice using antibodies directed against the C-terminus of Aβ 40 and Aβ 42 (lower panels). (c-e) Western blotting of Aβ PP and BACE1 activity assay. (c) Western blotting of AβPP in the cortex and thalamus of 17-month-old Ctr and Hpa-tg mice and a young adult Aβ PP KO mouse. (d) Quantification of the relative Aβ PP band intensities in homogenates from Ctr (n = 5) and Hpa-tg (n= 5) cortex and thalamus. (e) BACE1 activity assay of tissue lysates prepared from the cortex, hippocampus and thalamus of 17-month-old Ctr and Hpa-tg mice (n = 5). Data are expressed as ng of active BACE1/20 μg tissue.

**Additional file 6.** Heparanase immunostaining with pAb733 in AD hippocampus. The staining pattern reveals extensive Aβ deposit-like morphology. The framed region indicates the region from which the staining example presented in Fig. 3c is taken.
