# Supplementary material for: Heparanase overexpression impedes perivascular clearance of amyloid-β from murine brain: relevance to Alzheimer’s disease
Source: Acta Neuropathol Commun. 2021 May 10;9:84. doi: 10.1186/s40478-021-01182-x (PMC8111754; doi:10.1186/s40478-021-01182-x)
Supplement: Supplementary file 1 — Additional file 1. Supplementary Table 1: Primary antibodies used in this study. Supplementary Table 2: The middle temporalis gyrus of Alzheimer’s disease and Non-demented control obtained from the Netherlands Brain Bank (NBB). Supplementary Table 3: The CSF and plasma samples of Alzheimer´s disease patients and non-demented controls from the Netherlands Brain Bank (NBB). [file 40478_2021_1182_MOESM1_ESM.docx]

**Supplementary Table 1: Primary antibodies used in this study**

**Antibody Host Type Specificity Concentration** **Source**

| Anti-heparanase 733 | Rabbit | poly | Human, also mouse  50 kDa active enzyme | IHC: 1:250 | Vlodavsky lab |
| --- | --- | --- | --- | --- | --- |
| 6E10 | Mouse | mono | Human and rodent Aβ & AβPP | IHC:0.25µg/ml | Signet Lab. USA |
| Anti-human Aβ42  (anti-Aβ42) | Rabbit | poly | Human Aβ42  (C-terminal epitope specific) | IHC: 0.5µg/ml | BioSource, Belgium |
| Anti-human Aβ40  (anti-Aβ40) | Rabbit | poly | Human Aβ40  (C-terminal epitope specific) | IHC: 0.5µg/ml | BioSource, Belgium |
| 22C11 | Mouse | mono | Human and rodent AβPP | WB: 0.1µg/ml | Chemicon USA |
| Anti-rodent Aβ | Rabbit | poly | Rodent Aβ and AβPP | IHC, WB: 0.5µg/ml | abcam, UK |
| Anti-human Aβ [N-term] | Rabbit | poly | Human Aβ40, Aβ42 and Aβ43 (N-terminal epitope specific), cross reaction with rodent Aβ | IHC, DB: 1.0µg/ml | IBL Japan |
| Anti-vWF | Rabbit | poly | Mouse von Willebrand Factor | IHC: 0.5 µg/ml | Chemicon, USA |
| Anti-αSMA | Rabbit | poly | Human, mouse, rat  α smooth muscle actin | ICC: 1:100 | abcam, UK |
| Anti-Aquaporin 4 | Rabbit | poly | Human, mouse, rat | ICC: 1:100 | abcam, UK |
| Anti-GFAP | Mouse | mono | Human, mouse, rat  α smooth muscle actin | ICC: 1:100 | abcam, UK |
| Anti-GAPDH | Mouse | mono | Human and rodent  glyceraldehyde-3- phosphate dehydrogenase | WB: 0.1µg/ml | Santa Cruz, Germany |
| Anti-β-actin | Mouse | mono | Chicken, human, mouse, rat.  β-actin | WB: 0.1µg/ml | Invitrogen |
| Anti-β-Tubulin | Mouse | mono | Human, mouse, rat β-Tubulin | WB: 1µg/ml | Thermo Fisher |

IHC: Immunohistochemistry; ICC: Immunocytochemistry; WB: Western blotting; DB: dot blotting

**Supplementary table 2: The middle temporalis gyrus of Alzheimer’s disease and Non-demented control obtained from the Netherlands Brain Bank (NBB)**

|  |  | age | Braak | amyloid |
| --- | --- | --- | --- | --- |
| 1 | Alzheimer´s disease | 93 | 5 | C |
| 2 | Non-demented control | 93 | 1 | O |
|  |  |  |  |  |
| 3 | Alzheimer´s disease | 86 | 5 | C |
| 4 | Non-demented control | 89 | 1 | B |
|  |  |  |  |  |
| 5 | Alzheimer´s disease | 73 | 5 | C |
| 6 | Non-demented control | 73 | 2 | B |
|  |  |  |  |  |
| 7 | Alzheimer´s disease | 91 | 5 | B |
| 8 | Non-demented control | 89 | 2 | B |
|  |  |  |  |  |
| 9 | Alzheimer´s disease | 84 | 5 | C |
| 10 | Non-demented control | 84 | 1 | A |
|  |  |  |  |  |
| 11 | Alzheimer´s disease | 88 | 6 | C |
| 12 | Non-demented control | 82 | 2 | A |
|  |  |  |  |  |
| 13 | Alzheimer´s disease | 73 | 6 | C |
| 14 | Non-demented control | 76 | 1 |  |
|  |  |  |  |  |
| 15 | Alzheimer´s disease | 83 | 6 | C |
| 16 | Non-demented control | 78 | 1 | A |

**Supplementary table 3: The CSF and plasma samples of Alzheimer´s disease patients and non-demented controls from the Netherlands Brain Bank (NBB)**

| Alzheimer´s disease | age | Braak | amyloid | Non-demented control | age | Braak | amyloid |
| --- | --- | --- | --- | --- | --- | --- | --- |
| 1 | 87 | 5 | C | 1 | 99 | 2 | C |
| 2 | 74 | 5 | C | 2 | 82 | 3 | C |
| 3 | 87 | 6 | C | 3 | 92 | 4 | C |
| 4 | 78 | 5 | C | 4 | 95 | 3 | B |
| 5 | 83 | 6 | C | 5 | 102 | 3 | A |
| 6 | 85 | 6 | C | 6 | 88 | 3 | A |
| 7 | 85 | 4 | C | 7 | 91 | 3 | A |
| 8 | 88 | 4 | B | 8 | 89 | 3 |  |
| 9 | 78 | 5 | C | 9 | 92 | 3 | B |
| 10 | 86 | 6 | C | 10 | 93 | 2 | O |
| 11 | 81 | 6 | C | 11 | 60 | 0 |  |
| 12 | 82 | 5 | C | 12 | 96 | 3 |  |
| 13 | 73 | 5 | C | 13 | 88 | 4 |  |
| 14 | 76 | 6 | C | 14 | 82 | 2 | A |
| 15 | 94 | 4 | C |  |  |  |  |
| 16 | 86 | 5 | C |  |  |  |  |
| 17 | 93 | 6 | C |  |  |  |  |
| 18 | 85 | 6 | B |  |  |  |  |
| 19 | 85 | 6 | C |  |  |  |  |
| 20 | 87 | 5 | C |  |  |  |  |
| 21 | 84 | 5 | C |  |  |  |  |
| 22 | 85 | 5 | C |  |  |  |  |
| 23 | 85 | 5 | C |  |  |  |  |
| 24 | 84 | 5 | C |  |  |  |  |
| 25 | 86 | 5 | C |  |  |  |  |
| 26 | 84 | 5 | C |  |  |  |  |
| 27 | 84 | 5 | C |  |  |  |  |
