# Supplementary figures and images for: Heparanase overexpression impedes perivascular clearance of amyloid-β from murine brain: relevance to Alzheimer’s disease
Source: Acta Neuropathol Commun. 2021 May 10;9:84. doi: 10.1186/s40478-021-01182-x (PMC8111754; doi:10.1186/s40478-021-01182-x)

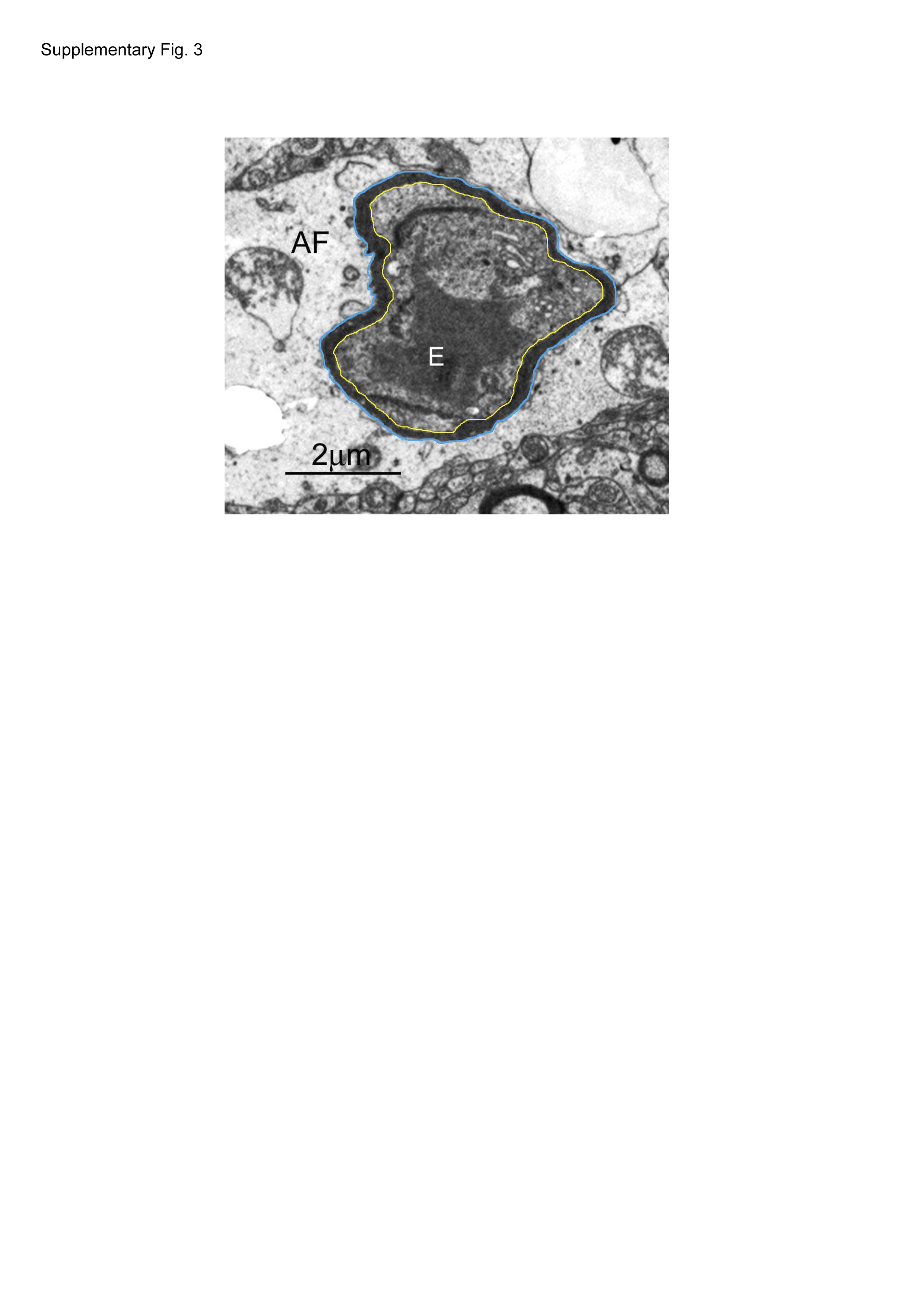

Supplement: Supplementary file 2 — Additional file 2. Thickness of vascular basement membrane (VBM). The VBM area was calculated using ImageJ software as the area enclosed by the blue curved line minus the area within the yellow curved line. Subsequently, the relative thickness of the VBM was calculated by dividing the VBM area by the capillary perimeter, i.e. the length of the blue curved line. The results were then expressed as BM thickness (nm) in Fig 2f. AF: astrocyte endfeet. E: erythrocyte. [file 40478_2021_1182_MOESM2_ESM.tif]

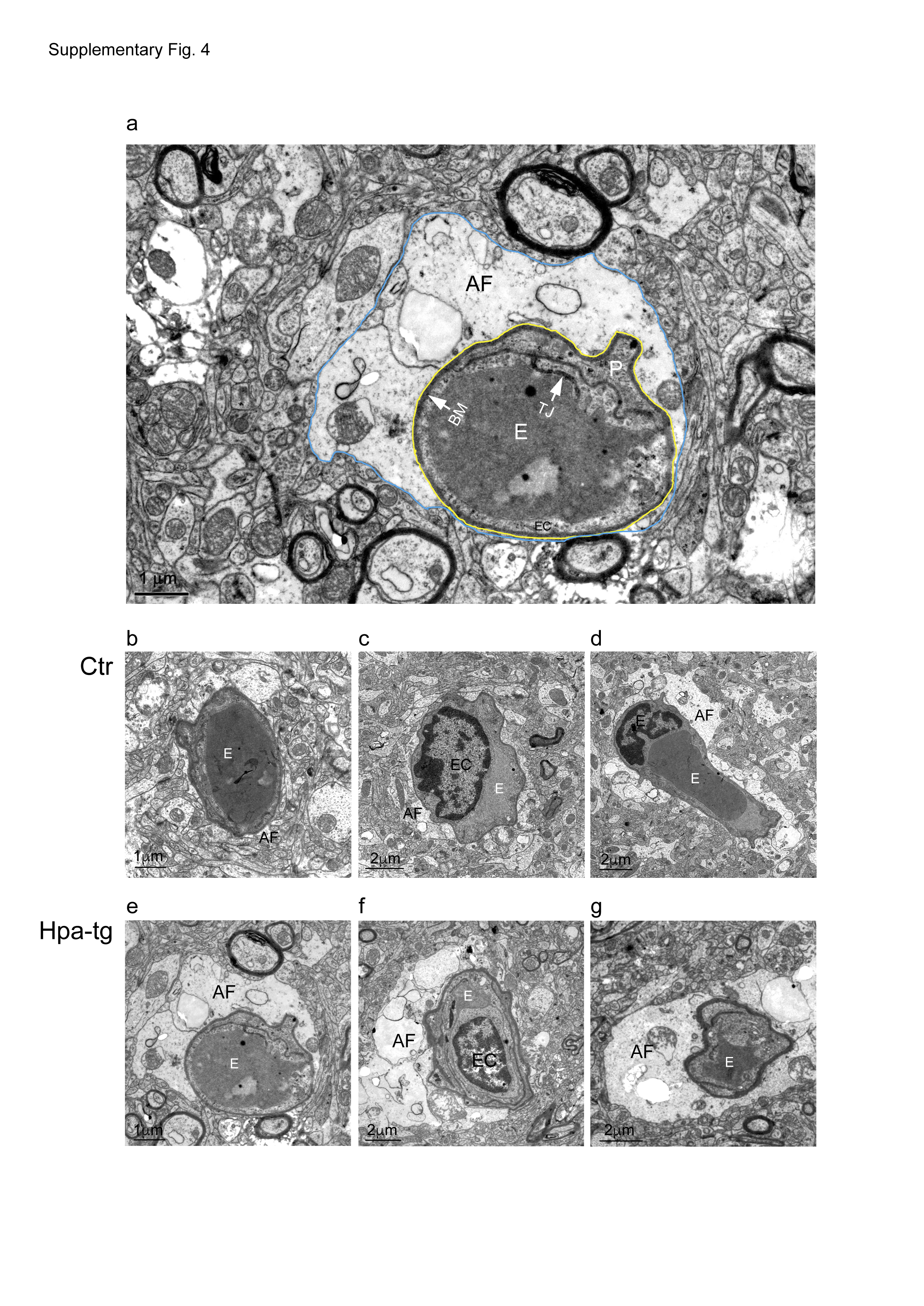

Supplement: Supplementary file 3 — Additional file 3. Swelling of astrocyte endfeet in Hpa-tg brain revealed by transmission electron microscopy. a) Image analysis was conducted using ImageJ software. The sums of the area of the astrocyte endfoot and the blood vessel-specific area were designated as the total capillary area, defined by the curved blue line. Thus the astrocyte endfoot area equaled the total capillary area minus the blood vessel-specific area (enclosed by the curved yellow line), which was then expressed as percentage of the total capillary area (Fig. 2g). b-g) Representative micrographs obtained from two mice of each strain (Hpatg and Ctr); b-d) Ctr. e-g) Hpa-tg. AF: astrocyte endfoot, BM: basement membrane, E: erythrocyte, EC: endothelial cell, TJ: tight junction. [file 40478_2021_1182_MOESM3_ESM.tif]

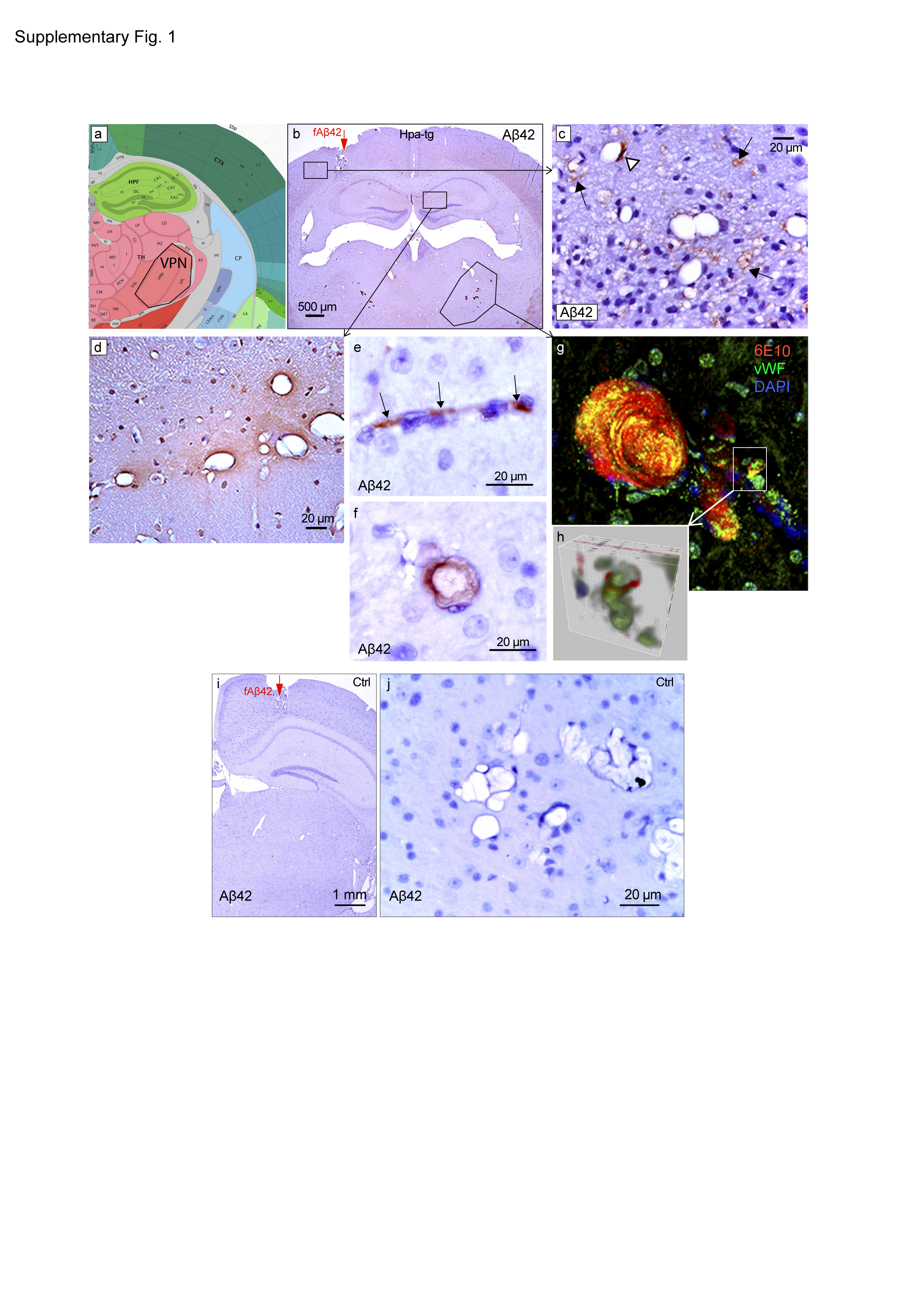

Supplement: Supplementary file 4 — Additional file 4. (a) Schematic illustration of mouse brain regions taken from the Allen Brain Atlas and identifying the location of the ventral posteromedial nucleus (VPL) and ventral posterolateral nucleus (VPM) of the thalamus, collectively referred to as ventral posterior nuclei (VPN). (b) Aβ42 immunostaining of a Hpa-tg brain section derived from a mouse that received an intracortical injection of fibrillar Aβ42 (fAβ42). (c) Enlarged view of a region close to the injection site in which Aβ42 was detected in interstitial spaces (arrows) and associated with blood vessels (arrowhead). (d) Enlarged view of a hippocampal region illustrating perivascular Aβ42 immunosignals. (e) Aβ42 immunosignals associated with cortical vasculature (arrows). (f) Aβ42 deposition in thickened VPN blood vessel wall. (g) Confocal microscopy of a thalamic deposit immunostained with anti-vWF for blood vessels (green) and the anti-Aβ antibody 6E10 (red). (h) Reconstructed three-dimensional rendering of one of the vessels in (g). (i) Aβ42 immunostaining of a control brain section from a mouse that had received an intracortical injection of fibrillar Aβ42 (fAβ42). (j) Enlarged view of the thalamic region in a Ctrl brain section. [file 40478_2021_1182_MOESM4_ESM.tif]

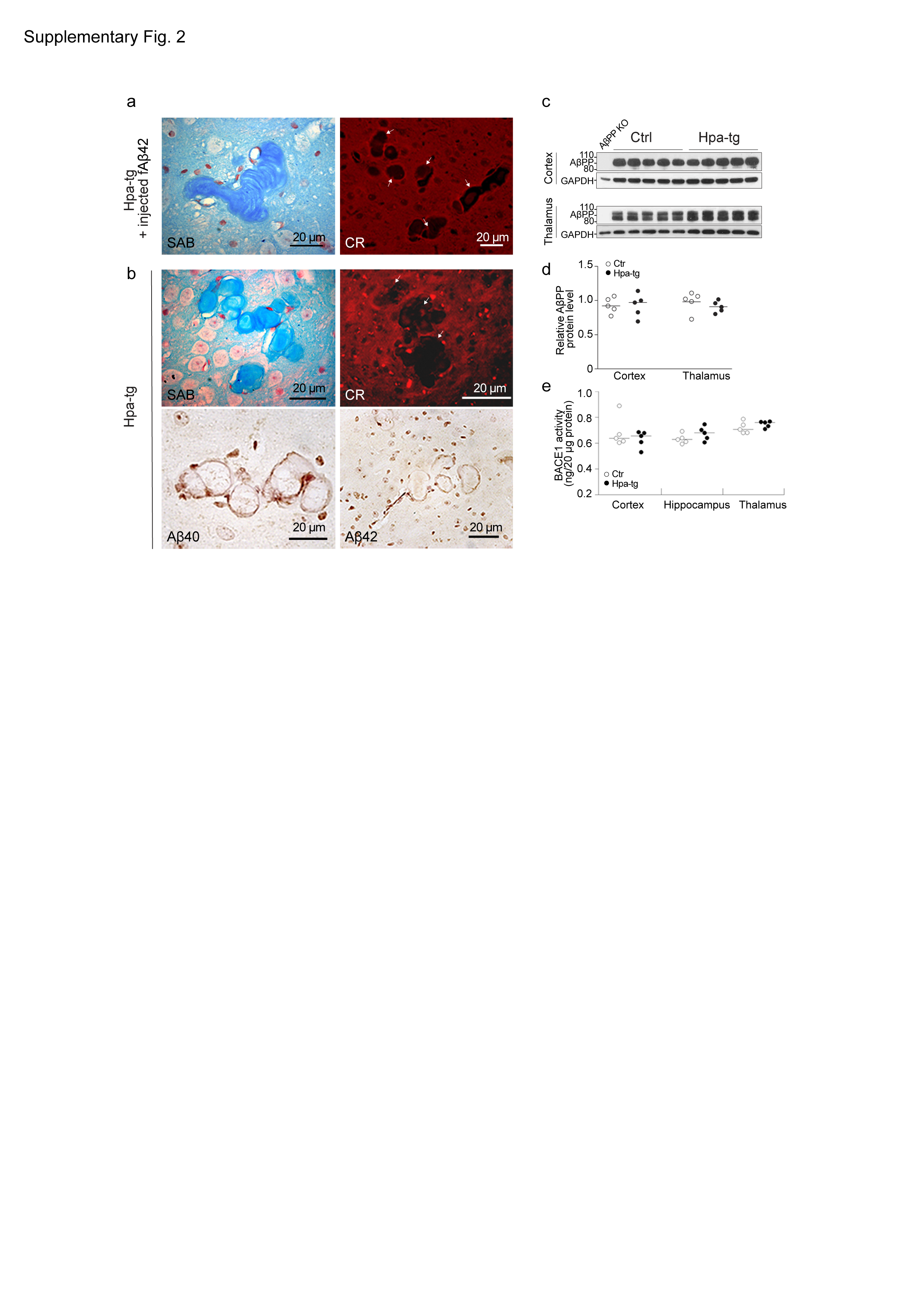

Supplement: Supplementary file 5 — Additional file 5. (a) Sulfated Alcian blue (SAB) and Congo red (CR) histochemical staining of the thalamic Aβ deposits in an Aβ-injected Hpa-tg mouse. (b) SAB and CR histochemical staining of the thalamic Aβ deposits in Hpa-tg brain sections from a mouse that had not been injected with Aβ (upper panels). Immunostaining of the thalamic structures in non-injected Hpa-tg mice using antibodies directed against the C-terminus of Aβ 40 and Aβ 42 (lower panels). (c-e) Western blotting of Aβ PP and BACE1 activity assay. (c) Western blotting of AβPP in the cortex and thalamus of 17-month-old Ctr and Hpa-tg mice and a young adult Aβ PP KO mouse. (d) Quantification of the relative Aβ PP band intensities in homogenates from Ctr (n = 5) and Hpa-tg (n= 5) cortex and thalamus. (e) BACE1 activity assay of tissue lysates prepared from the cortex, hippocampus and thalamus of 17-month-old Ctr and Hpa-tg mice (n = 5). Data are expressed as ng of active BACE1/20 μg tissue. [file 40478_2021_1182_MOESM5_ESM.tif]

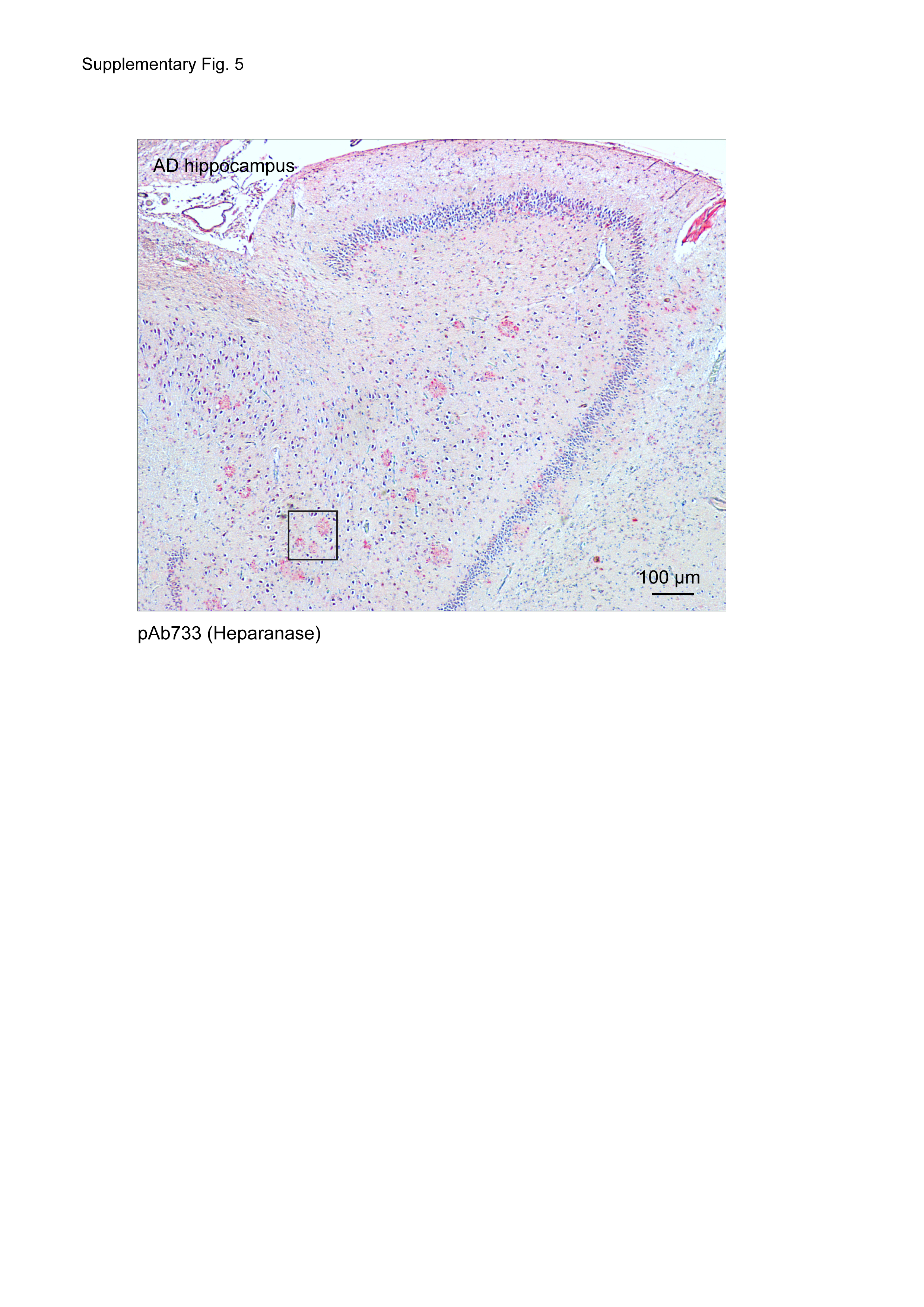

Supplement: Supplementary file 6 — Additional file 6. Heparanase immunostaining with pAb733 in AD hippocampus. The staining pattern reveals extensive Aβ deposit-like morphology. The framed region indicates the region from which the staining example presented in Fig. 3c is taken. [file 40478_2021_1182_MOESM6_ESM.tif]
